# Food Safety and Quality Interplay of Acrylamide Sensitive Products

**DOI:** 10.3390/foods15213800

**Published:** 2026-10-23

**Authors:** Bozidar Udovicki, Nevena Macanovic, Ilija Djekic

**Affiliations:** 1 Department of Food Safety and Quality Management, Faculty of Agriculture, University of Belgrade, Nemanjina 6, 11080 Belgrade, Serbia; idjekic@agrif.bg.ac.rs; 2 Faculty of Agriculture, University of Belgrade, Nemanjina 6, 11080 Belgrade, Serbia; nevenatosic.1997@gmail.com

**Keywords:** acrylamide, exposure assessment, consumer perception

## Abstract

Acrylamide is a neurotoxic and carcinogenic compound that forms in starch-rich food as a product of the Maillard reaction and is mostly associated with fried, baked, roasted, and toasted foods derived from cereals and potatoes. By employing two surveys, this study aimed to (i) estimate acrylamide dietary exposure in the Serbian adult population, characterize the risk of neurotoxic and neoplastic effects associated with acrylamide exposure, and (ii) investigate consumers’ perceptions of quality and food safety regarding commercially manufactured food products susceptible to acrylamide formation. The Monte Carlo simulation showed that mean acrylamide exposure among adult females ranged from 0.287 to 0.345 μg/kg bw per day, depending on the food intake survey method, while for adult males, mean exposure estimates obtained using the two food intake survey methods were very similar, ranging from 0.272 to 0.273 μg/kg bw per day. Conversely, the MOE values for neurotoxicity exceeded the risk-indicating threshold even at higher exposure percentiles, indicating no risk. The MOE values for neoplastic effects indicate a concern for both population groups, as they were well below the risk-indicating threshold. The highest confidence in the quality, safety, and healthiness of products susceptible to acrylamide formation was for nuts and legumes as well as for ready-to-eat and homemade products.

## 1. Introduction

Traditionally used as a monomer in the synthesis of polyacrylamides for industrial applications such as water treatment, papermaking, and soil conditioning, acrylamide did not become a food safety concern until 2002, when researchers discovered that it can form in food during heat processing. It forms in starch-rich food as a product of the Maillard reaction between the free amino acid asparagine and reducing sugars [1]. In addition to precursor compounds, high processing temperatures (typically above 120 °C) and low moisture content are key factors influencing acrylamide formation in food [2,3]. While several other minor pathways and precursors have been proposed for acrylamide formation, the Maillard reaction is considered the most important pathway. Acrylamide is most commonly associated with fried, baked, roasted, and toasted foods derived from cereals, beans, tubers, and storage roots, including bread, biscuits, breakfast cereals, potato chips, french/potato fries, roast potatoes, and coffee [4]. Acrylamide can form during frying, baking, and roasting, whether in domestic, industrial, or commercial settings.

The toxicological profile of acrylamide has been extensively investigated, with reported effects including neurotoxicity, genotoxicity, carcinogenicity, and reproductive toxicity [5]. The International Agency for Research on Cancer (IARC) has classified acrylamide as a Group 2A substance, i.e., “probably carcinogenic to humans”. It is also associated with cardiotoxicity, hepatotoxicity, immunotoxicity and developmental toxicity [6,7,8,9]. After recognizing acrylamide as a food-processing contaminant, the food industry, EU Member States, and the European Commission have conducted extensive research and mitigation efforts to characterize the pathways responsible for its formation and identify effective measures to limit its presence in processed foods. In 2017, Commission Regulation (EU) 2017/2158 was adopted, requiring food business operators to apply mitigation measures designed to keep acrylamide levels as low as reasonably achievable [10]. The Regulation also established benchmark levels used as performance indicators to assess whether mitigation measures are effective. These mitigation measures have reduced acrylamide concentrations in several food categories. However, they may have reached practical limits for certain foods, and achieving further substantial reductions is technically difficult without affecting product quality and sensory characteristics [11,12,13].

The study aimed to estimate dietary acrylamide exposure in the Serbian adult population, characterize the risk of neurotoxic and neoplastic effects associated with acrylamide exposure, and assess consumers’ perceptions of quality and food safety regarding commercially manufactured food products susceptible to acrylamide formation. While acrylamide exposure and associated health risks have been investigated to some extent in Serbia, there is still a need to expand the existing evidence. The novelty of the present study lies in including a larger number and wider variety of food samples, using collected food consumption data, and applying a probabilistic approach to dietary exposure assessment. By integrating these elements, the study aims to more comprehensively characterize dietary acrylamide exposure and its variability, supporting a more refined assessment of potential health risks. By examining the factors that determine food safety alongside consumers’ perceptions and evaluations of product quality, the study offers a novel perspective on the attributes that shape overall product assessment. The working hypothesis regarding consumers’ perceptions is that perceived quality, food safety, and the healthiness of this type of product positively influence consumers’ evaluations of the examined food products.

## 2. Materials and Methods

### 2.1. Online Surveys

The survey data were collected in Serbia during the second half of 2025 using two separate online questionnaires via the Google Forms^®^ platform: one assessing intake of commercially manufactured food products susceptible to acrylamide formation and the second assessing perceptions of quality and food safety of these food products. Only adults were included in these surveys, with adults defined as individuals aged 18–64 years [14]. This study used convenience sampling, recruiting participants who were accessible and available online. Because participation was open to anyone, the survey was unrestricted and self-selected, allowing individuals to decide whether to participate [15]. Respondents were recruited primarily through social media contacts and then disseminated further through various online channels. Before answering the questions, respondents were informed that the survey was anonymous and that they could leave the questionnaire at any time.

The initial target was to collect 700 respondents for each survey. A total of 683 respondents provided food intake data, while 691 respondents completed the consumer perception questionnaire; these answers were included in the final analysis. Given the size of the Serbian population, a 95% confidence level, and a 3.75% margin of error, the sample size is adequate [16,17]. Table 1 depicts the demographic characteristics of the samples.

#### 2.1.1. Food Consumption Survey

The food intake questionnaire was structured in three sections, as follows: (i) demographic characteristics (gender, age, body weight, education profile); (ii) Food Frequency Questionnaire (FFQ); (iii) 24 h recall-based food consumption survey. Both food intake methods covered the following ten food categories of commercially manufactured food products: biscuits and cookies; wafer biscuits; potato chips; bread and bakery products; extruded and pelleted snacks; tortilla chips; crackers and grissini (savoury snacks); muesli, granola and cornflakes; oatmeal and oat flakes; and peanuts. The semi-quantitative FFQ enabled participants to report both the frequency of consumption of each food item and the usual portion size consumed per eating occasion, allowing the estimation of the amount of each food consumed. The FFQ included the following food consumption frequency responses: 2 times per day, daily, 3–4 times per week, 2 times per week, weekly, 2–3 times per month, monthly, 1–6 times per year, and never. The quantity of consumed products was defined based on weight/number of pieces (biscuits and cookies, wafer biscuits, bread), the size of the retail package (potato and tortilla chips, extruded and pelleted snacks, crackers and grissini, peanuts) and common household measurements (cup of muesli, granola and cornflakes; oatmeal and oat flakes). The Supplementary Material Table S1 lists the portion sizes used. The 24 h recall-based food consumption survey used a simple food list approach, as explained previously [18,19,20,21]. Participants reported their food consumption during the previous 24 h using the same portion-size options as those provided in the FFQ, with an additional option to specify the amount consumed when it differed substantially from the predefined portion sizes. All participants completed both the FFQ and the 24 h recall food consumption survey. Because participants were recruited online and represented a convenience sample, we assessed 24 h dietary intake using a single-day 24 h dietary recall. Consequently, this method was used as a complementary assessment tool to the FFQ, providing further insight into the dietary practices and food consumption patterns of the Serbian population. The online questionnaires included visual aids, such as product photographs and defined portions. Details of the collected food intake data are in Supplementary Material Table S2.

#### 2.1.2. Perception Questionnaire

The questionnaire was developed based on similar research, integrating perspectives from various scientific manuscripts and adapted to the Serbian market and legislation [22,23,24,25,26]. Products were grouped into five categories in line with local legislation as follows: (i) fine bakery products (biscuits and tea pastries, waffle products/wafers, savoury snacks—grissini, pretzels, etc.); (ii) bakery products (bread, buns, bagels, scones, etc.); (iii) snacks (potato chips, flips, tortilla chips, etc.); (iv) ready-to-eat grain/cereal products (muesli/granola/cornflakes, oatmeal/oat flakes, etc.); (v) fried/baked nuts or legumes (peanuts). This approach may enhance the questionnaire’s relevance and support a more accurate assessment of consumer awareness of the quality and food safety aspects of foods susceptible to acrylamide formation.

The questionnaire was structured in four sections, as follows: (i) demographic characteristics (gender, age, education and employment status); (ii) general opinion towards the examined groups of products (selection of products that first come to the mind and whether consumption of this type of products increased or decreased in the last 12 months); (iii) 22 statements about this type of products allowing the respondents to rate their degree of agreement using a 5-point Likert scale (1—‘totally disagree’, 2—‘disagree’, 3—‘neutral’, 4—‘agree’, 5—‘totally agree’); (iv) most/least important quality characteristics and highest/lowest food safety hazards associated with this type of food (Table 2).

In line with the work of Merlino et al. (2018) [27], for this section, four attributes have been included in each subset (block), with a total of six blocks (for quality) and six blocks (for food safety). Blocks were constructed so that each attribute appeared four times across the design. Interviewees evaluated every block. We used a balanced incomplete block design to generate the choice sets, reducing cognitive burden while ensuring systematic coverage of comparisons, as shown in Supplementary Material Tables S3 and S4.

### 2.2. Occurrence of Acrylamide

Acrylamide in food products was determined by liquid chromatography–tandem mass spectrometry LC-MS/MS method (SRPS EN 16618:2015) in the accredited laboratory as part of the companies’ quality control procedure. The results used in the exposure assessment are presented in Table 3.

#### 2.2.1. Stock and Working Solution

Acrylamide (99.9%) (1 mg/mL) and acrylamide-d3 (1 mg/mL) (LGC; Dr Ehrenstorfer, Augsburg, Germany) stock and working standards were prepared in LC/MS-grade water: acetonitrile (75:25 *v*/*v*). To prepare working solutions, the stock solution was diluted with LC/MS-grade water.

#### 2.2.2. Acrylamide Extraction

One-gram samples from all foods were weighed in a 50 mL centrifuge tube. Then, 30 µL of acrylamide-d3 (10 µg/mL) was added, followed by vigorous shaking for 30 s and standing for 2 min. Subsequently, the following reagents were added sequentially, with shaking for 30–60 s after each addition: 5 mL of n-hexane (HPLC grade, VWR International, Rosny-sous-Bois, France), 10 mL of deionized water, and 10 mL of acetonitrile (HPLC grade, VWR International, Rosny-sous-Bois, France). The mixture was then shaken for 10 min using a horizontal shaker at a speed of >600 rpm.

For the first purification step, 3 g of the QuEChERS salt mixture (MgSO_4_/NaCl, 4 g/1 g) was carefully added (UCT Inc., Bristol, PA 19007, United States). The mixture was shaken vigorously for 1 min and then centrifuged for 5 min at 4000 rpm.

For the second purification step, 1 mL of the middle (acetonitrile) layer was transferred into a 2 mL dSPE tube containing 150 mg of anhydrous MgSO_4_, 50 mg of PSA, and 50 mg of C18 (UCT Inc., Bristol, PA 19007, United States). The tube was vortexed for 1 min and then centrifuged for 5 min at 4000 rpm. The resulting extract was filtered through a 0.22 µm membrane filter (13 mm diameter) into a 2 mL glass vial.

#### 2.2.3. Analysis Conditions

Liquid chromatographic analysis was performed using an ExionLC AD system (SCIEX, 250 Forest St, Marlborough, MA 01752, United States), equipped with an AD Autosampler (model SIL-20ACXR, Shimadzu Corporation, Kyoto, Japan) and a binary pump (model LC-20ADXR, Shimadzu Corporation, Kyoto, Japan), coupled to a QTRAP 5500 mass spectrometer (SCIEX, 250 Forest St, Marlborough, MA 01752, United States).

The analytical column used was a Brownlee Analytical C18 column (4.6 × 150 mm, 5 µm; PerkinElmer, Shelton, CT 06484, United States). The sample injection volume was 10 μL. The method was operated for 18 min using gradient elution with 0.1% formic acid in water (mobile phase A) and 0.1% formic acid in methanol (mobile phase B). Multiple reaction monitoring (MRM) was used for quantification, monitoring the transitions *m*/*z* 72 to 55 and *m*/*z* 72 to 27 for acrylamide, and *m*/*z* 75 to 58 and *m*/*z* 75 to 30 for acrylamide-d_3_. The flow rate was 0.8 mL/min. The column temperature was 30 °C. LC-MS/MS was operated on the positive ion electrospray. The ion spray voltage was set to 5500 V, and the ion source temperature was maintained at 550 °C. The curtain gas (CUR) was set to 30 psi, while gas 1 (GS1—nebulizing gas) and gas 2 (GS2—heater gas) were both set to 50 psi. The collision-activated dissociation (CAD) gas was set to medium.

#### 2.2.4. Calibration Curve and Analysis Validation

The calibration solutions were prepared using acrylamide stock solution and acrylamide-d3 stock solution with final concentrations (1, 5, 10, 30, 50, 100, 150 ng/mL for acrylamide and 30 ng/mL for acrylamide-d3). The calibration curve was created by adding 30 ng/mL acrylamide-d3 into seven different concentrations (1–150 ng/mL) using acrylamide stock solution. The prepared solutions were transferred to vials and read in an LC-MS/MS device. The calibration graph was plotted based on linear regression analysis of peak area of the acrylamide (Y) versus the concentration of acrylamide (X). Acrylamide concentrations in the sample extracts were calculated from the calibration curve by plotting the area ratio of acrylamide *m*/*z* 72 to that of acrylamide-d3 *m*/*z* 75 ions found in the sample. Linearity, limit of detection (LOD), and LOQ were evaluated to understand whether the LC-MS/MS method was suitable for analyzing acrylamide in samples. Based on the calibration range of 1–150 ng/mL, a sample mass of 1 g, and a solvent volume of 10 mL, the corresponding theoretical concentration range in the original samples was 10–1500 µg/kg. The limit of detection (LOD) of the method was determined to be 3 µg/kg.

The acrylamide content in the sample was calculated using the following equation:

(1) $$X\;=\;\frac{C*V}{m}$$

where X is the acrylamide content in the sample (ng/g, equivalent µg/kg), C is the concentration determined from the calibration curve (ng/mL), V is the solvent volume (mL), and m is the sample mass (g).

Method performance was evaluated through recovery experiments at five spiking levels (1, 5, 15, 35 and 75 ng/mL), corresponding to acrylamide concentrations of 10, 50, 150, 350 and 750 µg/kg in the original samples, based on a sample mass of 1 g and a solvent volume of 10 mL.

### 2.3. Exposure Assessment Model

The exposure to acrylamide was calculated as Estimated Daily Intake (EDI), separately for adult females and adult males, by multiplying the daily consumption for each food category with the corresponding occurrence levels and dividing the result by the individual’s body weight using a probabilistic methodology as described in detail previously [19,28,29].

The initial step involved fitting independent probability distributions for the consumption of each food category, acrylamide concentrations in the corresponding foods, and body weight for both populations; these were then used as probabilistic inputs and sampled to estimate the acrylamide EDI. The distributions were fitted using the “fitdistrplus” package. The most appropriate distributions were selected based on goodness-of-fit statistics, with particular emphasis on the Anderson–Darling statistic due to its relevance in risk assessment, as it gives greater consideration to the distribution tails while also accounting for the central part of the distribution [30,31,32]. All datasets were fitted as non-censored data [28,29], including concentration data with a very low percentage of samples without detected acrylamide (no detected or censored values). We replaced these negative values with the LOQ value to create an upper-bound scenario [33]. This substitution likely had minimal effect on the final EDI estimate because of the small number of negative samples and the difference between the LOQ and the mean acrylamide concentrations in the food samples. Supplementary Material Table S5 provides details of the fitted distributions for all datasets.

The fitted distributions for each dataset were randomly sampled using the “mc2d” package. A classical Monte Carlo simulation was performed nv times (nv = 1001) to account for variability in exposure, and the procedure was subsequently repeated nu times (nu = 1001) to account for parameter uncertainty [28,29,32]. This produced a two-dimensional nv × nu matrix from which the relevant statistics were derived. The variability and uncertainty incorporated in the preceding steps were propagated through the model outputs, with the EDI calculated using standard arithmetic operations. To account for negative values in food intake datasets (people who have not reported intake of specific items), the logical function “IF” was used [18,34].

Risk characterization was performed using Margin of Exposure (MOE) calculation, defined as the ratio between a toxicological reference point (a dose causing a low but measurable response) and the estimated intake [35] as presented in Equation (2). As the reference point, EFSA recommends using the BMDL_10_ (benchmark dose lower confidence limit at a 10% response level) [35].

(2) $$MOE=\frac{{BMDL}_{10}}{EDI}$$

To evaluate neurotoxicity risk, the EFSA CONTAM Panel recommended a BMDL_10_ of 0.43 mg/kg body weight (bw) per day as the reference point. In comparison, EFSA established a BMDL_10_ of 0.17 mg/kg body weight per day as the reference point for the risk characterization of neoplastic effects [5].

### 2.4. Statistical Processing of Perception Questionnaire

Selection of the first-mentioned products and demographic characteristics were analyzed using the chi-square test for association. Analysis of the first-mentioned product (within each group of products) and demographic characteristics were further subjected to correspondence analysis.

A principal component analysis (PCA) was conducted on 22 statements associated with foods susceptible to acrylamide formation. The suitability of the applied PCA was assessed before the analysis for the following criteria: analysis of the correlation coefficient in the correlation matrix, the value of the overall Kaiser–Meyer–Olkin measure and Bartlett’s test of sphericity. The statistical significance level was set at 0.05.

Most/least important quality characteristics and highest/lowest food safety hazards were analyzed using the Best-Worst score (Equation (3)), in line with the methodology outlined in similar food science research [36,37].

(3) $$BWS=\frac{B-W}{a\;\times \;n}$$

where B—frequency of being chosen as best (most important—highest hazard); W—frequency of being chosen as worst (least important—lowest hazard); a—availability in the series of six sets; n—number of respondents (n = 691).

In parallel, “Most-highest/Least-lowest percentages” (percentage of attributes chosen as “most-highest”, percentage of attributes chosen as “least-lowest”, and percentage of attributes not chosen) were also captured [38]. Both Best-Worst analyses were considered as ranks (“most-highest” was assigned “3”, “least-lowest” was assigned “1”, and if not selected was assigned “2”) and were subject to Friedman analysis followed by a comparison test for rank sums employing the Wilcoxon signed-rank test. Statistical significance was set at *p* < 0.05.

### 2.5. Statistical Software

Basic descriptive statistical analysis was performed in MS Excel (MS Office 365, Redmond, WA, USA). The two-dimensional Monte Carlo analysis, along with distribution fitting, was carried out in R: A Language and Environment for Statistical Computing (Version 4.6.1) using the “fitdistrplus” package (Version 1.1–8) and the “mc2d” package (Version 0.1–13). Minitab (Version 23) and SPSS (Version 17) were used to process the perception questionnaires. PCA analysis was performed with SPSS (Version 17).

## 3. Results and Discussion

### 3.1. Exposure Assessment and Risk Characterization

Table 4 and Table 5 present credible intervals of mean and percentile exposure levels across the two population groups and derived MOE values for neurotoxicity and neoplastic effects. For adult females, mean acrylamide exposure ranged from 0.287 to 0.345 μg/kg bw per day, depending on the food intake survey method. For adult males, mean exposure values from the two food intake survey methods were very close, ranging from 0.272 to 0.273 μg/kg bw per day. The 95th percentile exposure values were 0.868–0.901 and 0.666–0.828 μg/kg bw per day for adult females and males, respectively.

Mean MOE values for neurotoxicity ranged between 1246–1498 and 1575–1581 for adult females and males, respectively. For non-genotoxic compounds, an MOE of 100 and above is considered sufficient to conclude that there is no health concern [5]. The mean MOE values exceeded 100 in both population groups, indicating a low associated health concern. Even at the 95th and 99th percentiles, and within the credible interval, MOE values were above the threshold, indicating low risk concerns, even in the high-consumer segments of the population groups.

For genotoxic and carcinogenic substances, an MOE of 10,000 or higher is considered a lower level of concern from a public health perspective [5,39]. The MOE values for neoplastic effects observed in this study (Table 4 and Table 5) indicate a public health concern across dietary surveys, both population groups, and population percentiles, as they were well below the 10,000 threshold.

**Figure 1:**
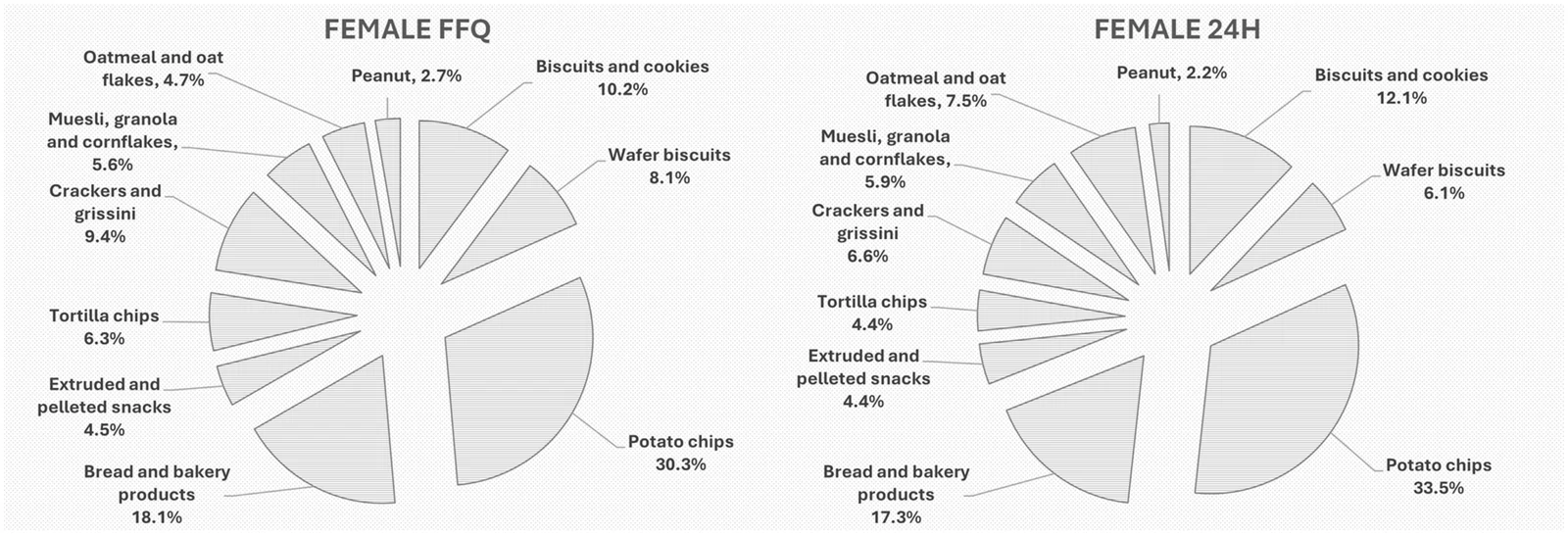
Contribution of eight food groups to acrylamide dietary exposure of adult females.

**Figure 2:**
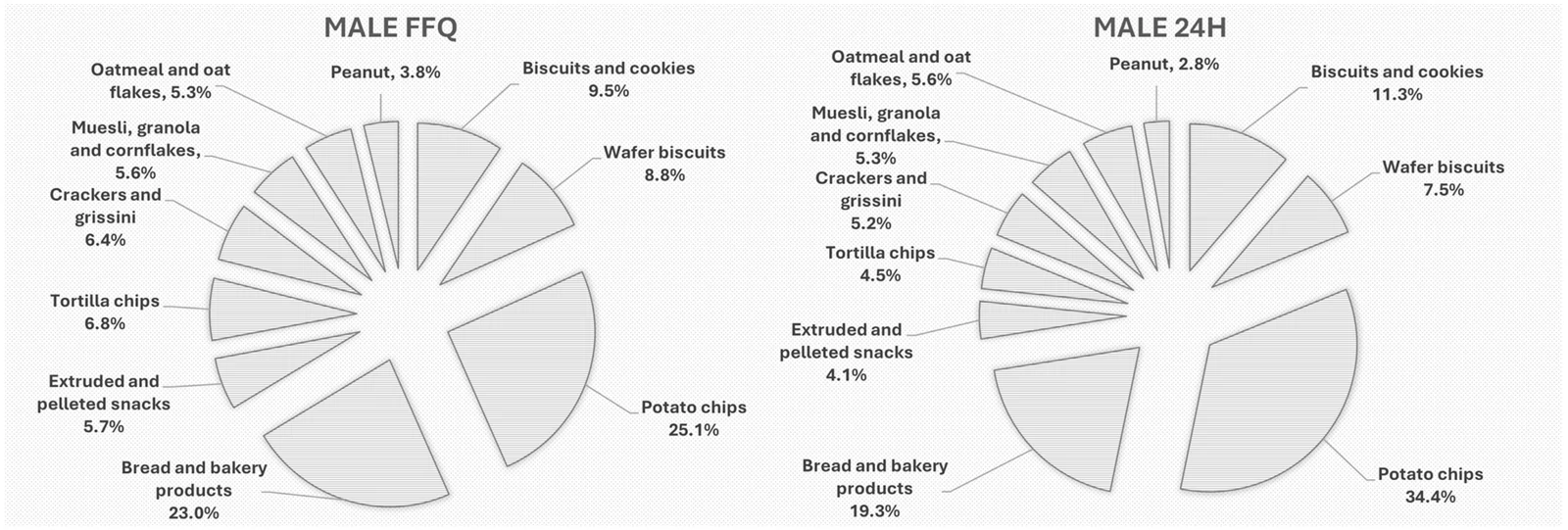
Contribution of eight food groups to acrylamide dietary exposure of adult males.

Figure 1 and Figure 2 show the contribution to acrylamide dietary exposure for eight food groups. Potato chips were the largest contributor, accounting for 25.1–34.4% of the total exposure. This was expected, as potato chips had the highest mean acrylamide level and is recognized as a major food source of acrylamide, along with potato fries [40]. The next highest contributors were bread and bakery products, at 17.3 to 23.0% of total exposure, reflecting higher and more regular consumption, as bread is a staple food in Serbia, with white flour bread consumption reaching up to three times the European average [41]. Among other food groups, biscuits and cookies contributed 9.5–12.1% of overall exposure, while all the remaining food groups contributed less than 10% each.

The results of this study can only be compared to a limited extent with those of other studies evaluating dietary acrylamide exposure, due to variations in food and population groups used for assessment and differences in methodological approaches.

In its exposure assessment, the Panel on Contaminants in the Food Chain (CONTAM) estimated the mean dietary exposure of the general adult European population to be in the range of 0.4–0.6 μg/kg bw per day, with the 95th percentile exposure ranging from 0.8 to 1.3 μg/kg bw per day [5]. Corresponding MOE values for mean exposure ranged between 717–1075 for neurotoxicity and 283–425 for neoplastic effects [5]. These values are slightly higher than results for the Serbian adult population. However, this study used a wider range of foods, including home-prepared foods. Across surveys, the main contributors were fried potato products (except potato chips); soft bread, biscuits, crackers, and crisp bread; and coffee, with most surveys showing contributions of 10–25%. Breakfast cereals, potato chips, and savoury snacks contributed up to 5% in most studies [5]. Peris-Camarasa et al. [3] investigated acrylamide exposure in Spanish and Italian adults from consuming potato chips, coffee, and crackers. The mean daily intake was 0.080 μg/kg bw per day for the Spanish population and 0.112 μg/kg bw per day for the Italian population, reaching 20–28% of the lowest EDI value reported by EFSA from the intake of just three foods. The resulting MOE values for neoplastic effects for the Spanish and Italian populations were 2126 and 1523, respectively, while MOE values for neurotoxicity were well above the threshold value [3]. Only one study has previously evaluated dietary exposure to acrylamide and associated health risks in Serbia, assessing individual food items and individual samples [42]. This study showed that consuming most bread samples and non-bread samples (biscuits, snacks, and potato chips) is associated with a risk indicating MOE for neoplastic effects. Among non-bread products, potato chips were the greatest concern for neoplastic effects. Basaran (2024) [43] estimated acrylamide exposure levels in the Turkish adult population (aged above 15 years) based on the consumption of nine food groups and reported EDI ranging from 0.85 μg/kg bw per day to 2.80 μg/kg bw per day, depending on the scenario. Depending on the scenario, potato fries, bread, black tea, and potato chips contributed most to overall exposure. A Croatian study from 2024 [44] revealed that the mean exposure of the adult population was in the range of 0.226–0.264 μg/kg bw per day, while exposure at the 95th percentile was in the range of 0.531–0.608 μg/kg bw per day for LB-UB scenarios, respectively. The MOE for neoplastic effects at mean exposure was 643, while the MOE for neurotoxicity at mean exposure was 1627. This study was based on the consumption of cereals and cereal-based products; coffee, cocoa, tea, and other infusions; composite dishes; legumes, nuts, oils, seeds, and spices; fruit and fruit products; and sugar, desserts, and similar products. The main contributors to acrylamide exposure were cereals and cereal-based products (mainly bread) at 69%, coffee, cocoa, tea, and other infusions at 14%, and composite dishes at 17% [44]. Exposure of the adult population of Cyprus was evaluated at 0.576 μg/kg bw per day based on a dietary survey conducted in 2014–2018 [45]. While the calculated MOE indicated potential concern about the neoplastic effects of acrylamide, the estimated dietary exposure levels did not suggest a concern for neurotoxicity. Fried potatoes (33%), rusks and crispbread (18%), and potato chips and biscuits (9%) contributed most to overall exposure [45]. A study aimed at determining the exposure of Polish adults (18–55 years) from traditional food consumption found mean and 95th percentile exposures of 0.213 and 0.458 μg/kg bw per day for the combined female and male population groups, respectively [46]. The MOEs for average exposure were 798 and 2019 for neoplastic effects and neurotoxicity, respectively. Mean acrylamide exposure of adult females was 0.208 μg/kg bw per day and adult males 0.220 μg/kg bw per day [46]. The assessment included traditional foods such as cookies, cheesecakes, salty sticks and shortbread snacks; bread, a local wheat bakery product; soft pretzels; bagels and pretzels; and pork paté and fish preserves. Specific foods contributed more evenly to the overall exposure than in other studies, with only bread and pork paté reaching a 15% contribution [46]. An early Finnish study [47] estimated acrylamide exposure in 25–44-year-old women at 0.44 μg/kg bw per day (median) and 1.16 μg/kg bw per day (97.5th percentile). In 25–44-year-old men, corresponding exposures were 0.41 μg/kg bw per day and 0.87 μg/kg bw per day. Among adults, coffee was the main source of acrylamide exposure, followed by starch-rich casseroles, and then rye bread [47].

Most of the reviewed studies reported results within or below the range reported by EFSA [5], which serves as a reference baseline for comparison in most studies. However, the food items investigated were not always the same. Although the data are not fully comparable across studies, certain patterns can still be identified both among previous studies and when their findings are compared with those of the present study. When included in dietary exposure assessments, fried potato is identified as a main source of acrylamide. Potato chips, the largest contributor to dietary acrylamide exposure in the present study, have also been identified as a major contributor in other studies. Similarly, bread and bakery products, the second-largest contributors in the present study, have also been recognized as important sources of dietary acrylamide in other populations. Biscuits and cookies contributed to overall acrylamide exposure in the Serbian population at levels comparable to those reported in some other countries. In contrast, crackers generally contributed a smaller proportion of total exposure in Serbia than in other populations. Similar patterns are observed globally, and most studies report that potato chips, fried potatoes, and other potato products contribute half or less than half of total acrylamide intake, and that other food items, such as breads and breakfast cereals, generally considered healthier, are also major contributors [48].

### 3.2. Consumer Perception Towards Products Susceptible to Acrylamide Formation

#### 3.2.1. General Opinion Towards the Examined Groups of Products

**Figure 3:**
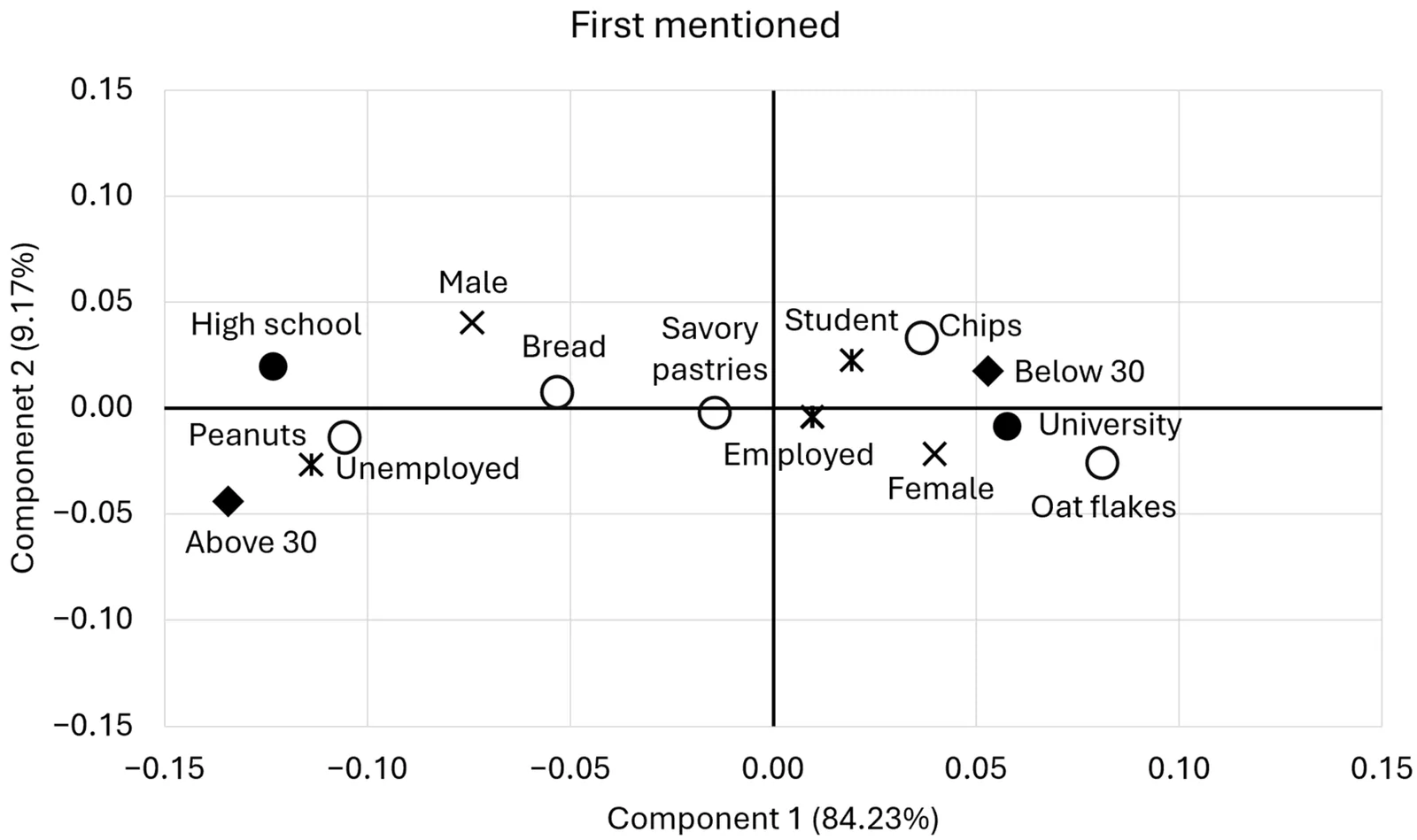
The biplot displays the results of the correspondence analysis based on the data collected from 691 respondents. Legend of symbols: ○ first mentioned products; × gender; ◆ age; ● education; ∗ employment status.

In this section, respondents answered two types of questions. First, they were asked to name the first product that came to mind for each product group. In each product group (Figure 3), one product clearly stood out, with shares between one and two thirds (savoury pastries—31.5%; bread—35.3%; peanuts—44.1%; oat flakes—59.2%; chips—59.5%).

The Chi-square test revealed a statistically significant association between selected fine bakery products and age, education, and work status (*p* < 0.05), with no statistically significant association for gender (*p* > 0.05). For bakery products, the same test showed a statistically significant association only with age (*p* < 0.05), with no statistically significant association with gender, education, or work status (*p* > 0.05). For snack products, the same test showed a statistically significant association with age, gender, and education (*p* < 0.05), with no statistically significant association with work status (*p* > 0.05). For ready-to-eat grain/cereal products, the Chi-square test showed a statistically significant association with all demographic characteristics (*p* < 0.05). Finally, for fried/baked nuts or legumes, age and work status showed a statistically significant association (*p* < 0.05), while gender and education did not (*p* > 0.05). This finding is consistent with similar studies showing that bakery product preferences vary across demographic groups [49,50,51].

The correspondence analysis shows that the first two dimensions explained 93.4% of the variance in the original contingency data. It shows that for the first group of products (fine bakery products), savoury pastries were mentioned first and are mainly linked to employed respondents. In the second group of products (bakery products), bread was mentioned first and was mainly associated with males and the high school-educated population. Potato chips was the first-mentioned snack product, clearly linked with students and younger respondents. In the fourth group (ready-to-eat grain/cereal products), oat flakes were mentioned most often and were associated with female and highly educated respondents. Finally, peanuts prevailed in the fifth group, linked with unemployed and older population.

Regarding the second question, whether consumption of a specific group of products increased, decreased, or stayed the same in the last 12 months, the prevailing answer for all product groups was “it stayed the same as 12 months ago”, with shares of 50.9% to 61.4%.

#### 3.2.2. Statements About Foods Susceptible to Acrylamide Formation

The correlation matrix from the Likert-scale statements showed that all variables had at least one correlation coefficient ≥ 0.35 (Table 6). The overall Kaiser-Meyer-Olkin measure was 0.81, and all individual KMO values exceeded 0.71, classifying them as ‘middling’/’meritorious’ [52]. Bartlett’s test of sphericity was statistically significant (*p* < 0.005), suggesting that the data were suitable for factor analysis. Eigenvalue analysis and the scree plot, along with the cut-off criterion of explaining over 60% of the total variance, indicated that four components should be retained [53]. Varimax orthogonal rotation was applied to improve interpretability.

Table 6 shows that Component 1—‘Nuts, legumes, ready-to-eat and homemade products’ has strong loadings for seven statements, and it represents consumers’ confidence on quality, safety and healthiness of fried/baked nuts or legumes and ready-to-eat grain/cereal products. It also presents consumers’ opinion about the high confidence in homemade products as well as the importance of information on food labels.

Component 2—‘Nutrition and colour’ has strong loadings with five statements reflecting consumers’ negative perception on the nutritional aspects (fat, salt, and sugar) of these products and highlighting their negative opinion on whether a darker colour of these products indicates safety and quality. The nutritional value of bakery products increasingly influences purchasing decisions [49], creating opportunities to promote health benefits through enriched products. Consumers perceive products enhanced with functional ingredients as beneficial [54].

Component 3—‘Bakery and fine bakery products’ has strong loadings for six statements, and it corresponds to consumers’ negative perception of the quality and healthiness of bakery and fine bakery products and their positive perception of their safety.

Component 4—‘Snack’ has strong loadings with three statements and brings together negative perception of the quality, safety and healthiness of snacks. This finding aligns with a U.S. study in which snacks—including cakes, cookies, pastries, and sweets—received the lowest nutritional profile scores [55].

The visual depiction of the quality-related B-W score (Table 7) shows that three of six quality characteristics are rated positively, with a statistically significant difference between the most and least important quality characteristics, χ^2^ = 666.595, *p* < 0.05. Further analysis (Wilcoxon signed-rank test) showed that there was a statistically significant difference between four quality characteristics: (i) overall taste; (ii) sweetness/saltiness and odour; (iii) texture; and (iv) appearance and colour. Respondents rated colour as the least important quality characteristic in over 50% of cases, which could be important when developing strategies to reduce the general population’s exposure to acrylamide. This finding aligns with Andaregie et al. (2024) [49], who identified taste as one of the most important consumer preferences at the point of purchase and colour as one of the least important. A study by Gębski et al. (2019) [54] confirms taste as the most important attribute in this type of product. One of the most basic strategies in both industrial and home cooking is achieving a lighter colour endpoint in the final product [10,56].

The depiction of the food safety-related B-W score (Table 8) shows that three of six food safety hazards are highly rated, with a statistically significant difference between the highest and lowest food safety hazards, χ^2^ = 560.719, *p* < 0.05. Further analysis (Wilcoxon signed-rank test) showed that there was a statistically significant difference between three groups of food safety hazards: (i) microbial and chemical hazards; (ii) nutritional composition and physical hazards; (iii) type of packaging and food label. The main two types of hazards identified by the respondents align with scientific studies. Smith et al. (2004) [57] discuss microbial risks such as microbiological spoilage by bacteria, yeast and moulds in high moisture bakery products. In addition to acrylamide, these foods may contain other chemical hazards—including furan derivatives, polycyclic aromatic amines, glycidol, and their esters—formed through the Maillard reaction during baking, roasting, steaming, and frying [58].

## 4. Conclusions

The results of the present study indicate no concern regarding the neurotoxic effects of acrylamide, whereas the MOE values for neoplastic effects indicate a potential health concern for both population groups. This potential concern should not be overlooked, given acrylamide’s established carcinogenic potential. Although estimated exposure was lower than the European average, this study used commercially manufactured food products and did not include major contributors from home and restaurant meals, most notably potato fries and baked potatoes. It is reasonable to hypothesize that full exposure would match the European level. Given that, for some food products susceptible to acrylamide formation, current mitigation measures may have reached their practical limits, further reductions in dietary exposure may require reducing the consumption of these products. Following general recommendations to limit ultra-processed foods and snacks could help reduce acrylamide exposure, as these products contribute significantly to dietary acrylamide exposure in the Serbian adult population. However, dietary exposure to acrylamide is not limited to foods commonly perceived as unhealthy, as staple foods such as bread may also contribute substantially to overall intake. Therefore, public awareness should first be raised regarding the presence of acrylamide in food, followed by a better understanding that exposure is not limited to foods commonly perceived as unhealthy. Raising awareness should also encourage consumers to make informed decisions about their diets and food preparation methods, while supporting research initiatives aimed at reducing acrylamide formation at food preparation and production stages. This study confirms the working hypothesis that perceived quality, food safety, and healthiness positively shape consumers’ evaluations of nuts, legumes, ready-to-eat foods, and homemade products, but negatively shape their perceptions of bakery products, fine bakery products, and snacks.

## Figures and Tables

**Table 1:** Demographic characteristics of the tested populations.

Food Intake Survey		Perception Survey	
	**Total N (%)**		**Total N (%)**
**Gender**		**Gender**	
Male	275 (40.3%)	Male	232 (33.6%)
Female	408 (59.7%)	Female	459 (66.4%)
**Age**		**Age**	
Below 30 yrs	524 (76.7%)	Below 30 yrs	446 (64.5%)
Above 30 yrs	159 (23.3%)	Above 30 yrs	245 (35.5%)
**Residence**		**Education**	
Urban	505 (73.9%)	High school graduates	238 (34.4%)
Suburban and rural	178 (26.1%)	Bachelor’s degree holders and beyond	453 (65.6%)
Body mass index *		**Employment status**	
Underweight (BMI < 18.5)	24 (3.5%)	Unemployed	77 (11.1%)
Healthy weight (18.5 < BMI ≤ 24.9)	435 (62.7%)	Student	154 (22.3%)
Overweight and obese (BMI > 25.0 and >30)	224 (32.8%)	Employed	460 (66.6%)

Legend: N represents the number of interviewees; (%) represents their share in the sample. *—The average body weight of the tested population was 64.5 kg and 87.4 kg for adult females and adult males, respectively.

**Table 2:** Attributes used as quality characteristics/food safety hazards in the Best-Worst analysis.

Quality Characteristics	Food Safety Hazards
Overall taste	Chemical hazard
Appearance	Microbial hazard
Colour	Physical hazard
Sweetness/saltiness	Type of packaging
Texture	Food label
Odour	Nutritional composition

**Table 3:** The occurrence of acrylamide in analyzed products.

Product	N	Np	Mean LB (µg/kg)	Mean UB (µg/kg)	Median (µg/kg)	Min (µg/kg)	Max (µg/kg)
Biscuits and cookies	126	126	172.3	172.3	167.8	24.1	682.7
Wafer biscuits	100	100	237.7	237.7	228.1	48.3	502.3
Potato chips	213	213	589.3	589.3	569.4	72.4	2005.0
Bread and bakery products	48	44	59.0	59.9	42.2	11.1	291.7
Extruded and pelleted snacks	73	72	99.2	99.4	100.7	24.8	293.0
Tortilla chips	82	80	172.7	173.0	145.8	19.0	567.4
Crackers and grissini	69	68	197.7	197.8	147.1	22.4	1047.7
Muesli, granola and cornflakes	118	115	109.7	109.9	93.0	10.8	416.5
Oatmeal and oat flakes	24	20	89.1	90.8	42.1	22.4	450.6
Peanut	22	22	81.9	81.9	81.3	22.8	187.7
**Total**	**875**	**860**					

Legend: N—number of samples; Np—number of positive samples; LB (Lower bound)—non-detects were replaced with 0; UB (Upper bound)—non-detects were replaced with the value of the Limit of Quantification (LOQ = 10 µg/kg).

**Table 4:** Acrylamide exposure (μg/kg bw/day) and risk characterization in adult females.

Female FFQ							Female 24 h						
**EDI**	**Mean**	**25th**	**50th**	**75th**	**95th**	**99th**	**EDI**	**Mean**	**25th**	**50th**	**75th**	**95th**	**99th**
**Median**	0.342	0.152	0.246	0.401	0.864	1.710	**Median**	0.286	0.075	0.188	0.386	0.899	1.440
**Mean**	0.345	0.152	0.247	0.402	0.868	1.760	**Mean**	0.287	0.075	0.188	0.388	0.901	1.450
**2.5th**	0.302	0.138	0.223	0.358	0.731	1.280	**2.5th**	0.260	0.061	0.163	0.345	0.792	1.210
**97.5th**	0.399	0.170	0.275	0.453	1.040	2.510	**97.5th**	0.317	0.090	0.216	0.435	1.019	1.730
**MOE** **neurotoxicity**	**Mean**	**25th**	**50th**	**75th**	**95th**	**99th**	**MOE** **neurotoxicity**	**Mean**	**25th**	**50th**	**75th**	**95th**	**99th**
**Median**	1257	2829	1748	1072	498	251	**Median**	1503	5756	2287	1114	478	299
**Mean**	1246	2829	1741	1070	495	244	**Mean**	1498	5741	2287	1108	477	297
**2.5th**	1424	3116	1928	1201	588	336	**2.5th**	1654	7038	2638	1246	543	355
**97.5th**	1078	2529	1564	949	413	171	**97.5th**	1356	4794	1991	989	422	249
**MOE** **neoplastic**	**Mean**	**25th**	**50th**	**75th**	**95th**	**99th**	**MOE** **neoplastic**	**Mean**	**25th**	**50th**	**75th**	**95th**	**99th**
**Median**	497	1118	691	424	197	99	**Median**	594	2276	904	440	189	118
**Mean**	493	1118	688	423	196	97	**Mean**	592	2270	904	438	189	117
**2.5th**	563	1232	762	475	233	133	**2.5th**	654	2782	1043	493	215	140
**97.5th**	426	1000	618	375	163	68	**97.5th**	536	1895	787	391	167	98

The distribution of the EDI and MOE values (variability dimension of the population exposure) is presented in rows. The uncertainty dimension is presented in columns as the median, mean, and credible interval of EDI and MOE means and percentiles.

**Table 5:** Acrylamide exposure (μg/kg bw/day) and risk characterization in adult males.

Male FFQ							Male 24 h						
**EDI**	**Mean**	**25th**	**50th**	**75th**	**95th**	**99th**	**EDI**	**Mean**	**25th**	**50th**	**75th**	**95th**	**99th**
**Median**	0.271	0.132	0.212	0.339	0.665	1.088	**Median**	0.273	0.077	0.187	0.376	0.824	1.290
**Mean**	0.272	0.132	0.213	0.339	0.666	1.097	**Mean**	0.273	0.077	0.187	0.376	0.828	1.290
**2.5th**	0.241	0.117	0.190	0.301	0.573	0.874	**2.5th**	0.246	0.063	0.163	0.334	0.730	1.070
**97.5th**	0.304	0.146	0.236	0.379	0.782	1.403	**97.5th**	0.303	0.091	0.213	0.420	0.941	1.540
**MOE** **neurotoxicity**	**Mean**	**25th**	**50th**	**75th**	**95th**	**99th**	**MOE** **neurotoxicity**	**Mean**	**25th**	**50th**	**75th**	**95th**	**99th**
**Median**	1587	3258	2028	1268	647	395	**Median**	1575	5584	2299	1144	522	333
**Mean**	1581	3258	2019	1268	646	392	**Mean**	1575	5577	2299	1144	519	333
**2.5th**	1784	3675	2263	1429	750	492	**2.5th**	1748	6836	2638	1287	589	402
**97.5th**	1414	2945	1822	1135	550	306	**97.5th**	1419	4705	2019	1024	457	279
**MOE** **neoplastic**	**Mean**	**25th**	**50th**	**75th**	**95th**	**99th**	**MOE** **neoplastic**	**Mean**	**25th**	**50th**	**75th**	**95th**	**99th**
**Median**	627	1288	802	501	256	156	**Median**	623	2208	909	452	206	132
**Mean**	625	1288	798	501	255	155	**Mean**	623	2205	909	452	205	132
**2.5th**	705	1453	895	565	297	195	**2.5th**	691	2703	1043	509	233	159
**97.5th**	559	1164	720	449	217	121	**97.5th**	561	1860	798	405	181	110

The distribution of the EDI and MOE values (variability dimension of the population exposure) is presented in rows. The uncertainty dimension is presented in columns as the median, mean, and credible interval of EDI and MOE means and percentiles.

**Table 6:** Rotated structure matrix for the principal component analysis with Varimax rotation with four components.

	Component				Av ± SD|M *
	1	2	3	4	
Fried/baked nuts or legumes are healthy food	**0.843**	−0.004	0.060	−0.075	3.63 ± 1.07|4
Ready-to-eat grain/cereal products are safe food	**0.836**	0.018	0.073	0.076	3.63 ± 1.01|4
Fried/baked nuts or legumes are safe food	**0.827**	0.009	0.030	0.095	3.65 ± 0.99|4
Fried/baked nuts or legumes are quality food	**0.810**	0.036	0.028	0.041	3.52 ± 1.05|4
Ready-to-eat grain/cereal products are healthy food	**0.782**	0.100	0.075	−0.002	3.62 ± 1.03|4
Ready-to-eat grain/cereal products are quality food	**0.705**	0.156	−0.009	0.107	3.34 ± 1.03|4
I have more confidence in homemade products than industrial products	**0.595**	−0.061	0.053	−0.342	3.92 ± 1.18|5
I pay attention to the information on the food labels when purchasing these products	**0.378**	−0.025	0.018	−0.103	3.36 ± 1.17|4
Although some of these products are rich in salt, their consumption does not contribute to obesity	−0.003	**0.782**	0.110	0.166	2.16 ± 1.03|2
Although some of these products are rich in fat, their consumption does not contribute to obesity	0.028	**0.774**	0.136	0.079	2.32 ± 1.12|2
Although some of these products are rich in sugar, their consumption does not contribute to obesity	0.095	**0.738**	0.050	0.046	2.07 ± 1.02|2
Darker colour of product indicated higher quality	0.014	**0.714**	0.162	0.027	2.21 ± 0.91|2
Darker colour of product indicated a safer product	0.075	**0.700**	0.177	0.007	2.18 ± 0.91|2
Bakery products are quality food	0.060	0.163	**0.805**	0.030	2.19 ± 0.92|2
Bakery products are healthy food	0.037	0.215	**0.787**	−0.019	2.13 ± 0.92|2
Fine bakery products are quality food	−0.010	0.267	**0.633**	0.250	1.96 ± 0.78|2
Fine bakery products are healthy food	−0.064	0.298	**0.631**	0.133	1.86 ± 0.79|2
Bakery products are safe food	0.297	−0.125	**0.621**	**0.416**	3.01 ± 1.09|4
Fine bakery products are safe food	0.252	−0.144	**0.548**	**0.495**	2.98 ± 1.06|4
Snacks are safe food	0.154	−0.053	0.237	**0.789**	2.40 ± 1.13|2
Snacks are healthy food	−0.254	0.301	0.078	**0.710**	1.71 ± 0.85|1
Snacks are quality food	−0.208	**0.383**	0.188	**0.692**	1.77 ± 0.86|1

* Av—average value, SD—standard deviation; M—mode. 5-point Likert scale: 1—‘totally disagree’, 2—‘disagree’, 3—‘neutral’, 4—‘agree’, 5—‘totally agree’.

**Table 7:** Subjective priority of most and least important quality characteristics. Best-Worst scaling report: frequency counts and standardized average score considering the entire sample (N = 691). Legend: Values marked with different letter are statistically different.

**Attributes**	**Number of “Most Important”**	**Number of “Least Important”**	**Distribution [%]**			**Share of Preference [%]**	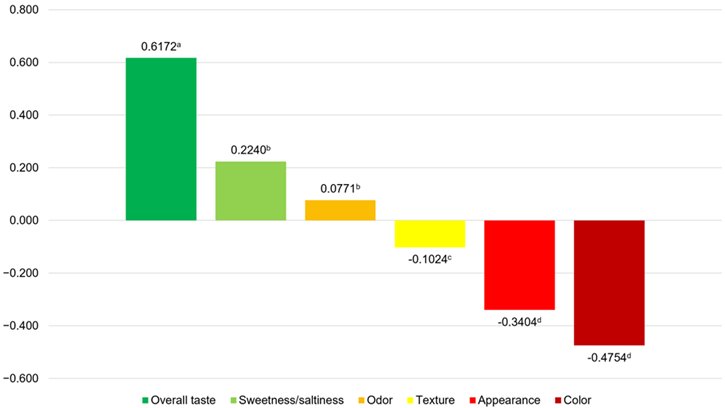
			**Most Important**	**Least Important**	**Not Chosen**		
Overall taste	1888	182	68.31%	6.58%	25.11%	45.54%	
Sweetness/saltiness	913	294	33.03%	10.64%	56.33%	22.02%	
Odour	495	282	17.91%	10.20%	71.89%	11.94%	
Texture	402	685	14.54%	24.78%	60.67%	9.70%	
Appearance	261	1202	9.44%	43.49%	47.07%	6.30%	
Colour	187	1501	6.77%	54.31%	38.93%	4.51%	

**Table 8:** Subjective priority of highest and lowest food safety hazards. Best-Worst scaling report: frequency counts and standardized average score considering the entire sample (N = 691). Legend: Values marked with different letter are statistically different.

**Attributes**	**Number of “Highest Hazard”**	**Number of “Lowest Hazard”**	**Distribution [%]**			**Share of Preference [%]**	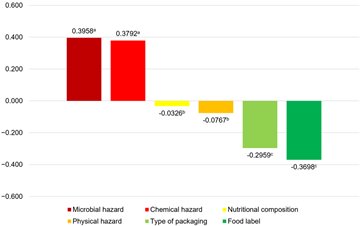
			**Highest Hazard**	**Lowest Hazard**	**Not Chosen**		
Microbial hazard	1377	283	49.82%	10.24%	39.94%	33.21%	
Chemical hazard	1329	281	48.08%	10.17%	41.75%	32.05%	
Nutritional composition	466	556	16.86%	20.12%	63.02%	11.24%	
Physical hazard	370	582	13.39%	21.06%	65.56%	8.92%	
Type of packaging	299	1117	10.82%	40.41%	48.77%	7.21%	
Food label	305	1327	11.03%	48.01%	40.96%	7.36%	

## Data Availability

The original contributions presented in this study are included in the article/Supplementary Materials. Further inquiries can be directed to the corresponding author.

## Supplementary Materials

The following supporting information can be downloaded at: https://www.mdpi.com/article/10.3390/foods15213800/s1, Table S1: Used portion sizes; Table S2: Average daily intake of food products (g); Table S3. Balanced incomplete block (BIB) model used in the survey about quality characteristics; Table S4. Balanced incomplete block (BIB) model used in the survey about food safety hazards; Table S5: Distribution fitted to the intake, concentration, and bodyweight data sets.
