# Supplementary material for: Food Safety and Quality Interplay of Acrylamide Sensitive Products
Source: Foods. 2026 Oct 23;15(21):3800. doi: 10.3390/foods15213800 (PMC13763038; doi:10.3390/foods15213800)
Supplement: Supplementary file 1 [file foods-15-03800-s001.zip › foods-4618628-supplementary.pdf]

Table S1. Used portion sizes

Product	Portion				
**Biscuits and cookies**	About 25g (2 pieces)	About 50g (4 pieces)	About 75g (6 pieces)	About 100g (8 pieces)	About 125g (10 pieces)
**Wafer biscuits**	About 25g (2 pieces)	About 50g (4 pieces)	About 75g (6 pieces)	About 100g (8 pieces)	About 125g (10 pieces)
**Potato chips**	About 20g (1/2 of small package)	About 40g (small package)	About 60g (medium package)	About 90g (large package)	
**Bread and bakery products**	About 30g (1 slice)	About 60g (2 slices/ 1 bread roll)	About 90g (3 slices/ 1 croissant)	About 120g (4 slices / 2 bread rolls)	About 150g (5 slices)
**Extruded and pelleted snacks**	About 30g (small package)	About 50g (medium package)	About 80g (large package)	About 100g (extra-large package)	
**Tortilla chips**	About 30g (1/2 of medium package)	About 60g (medium package)	About 90g (large package)	About 150g (extra-large package)	
**Crackers and grissini**	About 20g (1/2 of small package)	About 40g (small package)	About 90g (large package)		
**Muesli, granola and cornflakes**	About 60g (1⁄2 of 200ml cup)	About 90g (2/3 of 200ml cup)	About 120g (200ml cup)	About 180g (1&1/2 of 200ml cup)	
**Oatmeal and oat flakes**	About 60g (1⁄2 200ml cup)	About 90g (2/3 of 200ml cup)	About 120g (200ml cup)	About 180g (1&1/2 of 200ml cup)	
**Peanut**	About 20g (1/2 of small package)	About 40g (small package)	About 60g (medium package)	About 90g (large package)	

Table S2. Average daily intake of food products (g)

	Female FFQ	Female 24h	Male FFQ	Male 24h
**Biscuits and cookies**	13.2	12.6	12.7	15.1
**Wafer biscuits**	7.5	4.7	8.8	7.4
**Potato chips**	9.4	10.3	10.0	13.5
**Bread and bakery products**	65.4	51.5	89.7	74.0
**Extruded and pelleted snacks**	9.9	8.1	11.1	9.6
**Tortilla chips**	8.2	4.6	9.3	6.1
**Crackers and breadsticks**	8.7	5.9	7.5	6.1
**Muesli, granola and cornflakes**	11.3	9.7	10.0	11.1
**Oatmeal and oat flakes**	11.6	14.9	11.3	14.1
**Peanut**	7.6	4.8	10.7	7.9

Table S3. Balanced incomplete block (BIB) model used in the survey about quality characteristics

P = 6 the number of evaluated quality characteristics (attributes).

k = 4 the number of quality characteristics (attributes) in a single block.

r = 4 the number each quality characteristic (attribute) appears in the model.

b = 6 the number of blocks in the model.

m = 691 the number the BIB model was repeated (in this case this is the number of participants n)

**Blocks**	**Quality characteristics**					
	**A**	**B**	**C**	**D**	**E**	**F**
1		B		D	E	F
2	A		C	D	E	
3	A	B	C			F
4	A	B			E	F
5		B	C	D	E	
6	A		C	D		F

Table S4. Balanced incomplete block (BIB) model used in the survey about food safety hazards

P = 6 the number of evaluated food safety hazards (attributes).

k = 4 the number of food safety hazards (attributes) in a single block.

r = 4 the number each food safety hazard (attribute) appears in the model.

b = 6 the number of blocks in the model.

m = 691 the number the BIB model was repeated (in this case this is the number of participants n)

**Blocks**	**Food safety hazards**					
	**A**	**B**	**C**	**D**	**E**	**F**
1		B		D	E	F
2	A		C	D	E	
3	A	B	C			F
4	A	B			E	F
5		B	C	D	E	
6	A		C	D		F

Table S5. Distribution fitted to the intake, concentration, and bodyweight data sets

	Female FFQ	Female 24h	Male FFQ	Male 24h	Acrylamide	Female bw	Male bw
**Biscuits and cookies**	weibull	weibull	weibull	weibull	weibull	lognorm al	lognormal
**Wafer biscuits**	weibull	weibull	weibull	gamma	weibull		
**Potato chips**	lognormal	gamma	weibull	weibull	gamma		
**Bread and bakery products**	beta	weibull	beta	weibull	lognormal		
**Extruded and pelleted snacks**	weibull	lognormal	lognormal	gamma	weibull		
**Tortilla chips**	weibull	lognormal	weibull	weibull	gamma		
**Crackers and breadsticks**	lognormal	weibull	weibull	lognormal	gamma		
**Muesli, granola and cornflakes**	weibull	weibull	lognormal	lognormal	gamma		
**Oatmeal and oat flakes**	weibull	beta	lognormal	lognormal	weibull		
**Peanut**	weibull	gamma	weibull	beta	gamma		
